# The Results of Nerve Injury

**Published:** 1877-04

**Authors:** 


### ARTICLE XI.

### *The Results of Nerve Injury.*

Mr. Gay, of the Northern Hospital, London, in a recent clinical lecture, reported in the *Lancet* spoke as follows:

The results of nerve injury from accidents and operations are interesting. The division of considerable nerve-branches —such as (1) those we are more familiar with—viz., the ulnar and the pereoneal, (2) of smaller branches, filaments, or twigs, near their peripheral termination, as in ordinary operation or wounds or in skin-lifting, and (3) of sympathetic branches, as by experiment is followed by curious results. In the first, the parts to which the divided nerve is distributed may suffer permanent or only temporary loss of nerve power, or that power may be partially recovered; in the second, in the course of severe wound, such as that just related, no results attribulate to nerve lesion can usually be detected; whilst in skin shifting the effects of nerve lesion may or may not follow. In some instances, as in those about the neck, when not very extensive, they do not; neither do they usually about the face; but in the shifting of flaps, such as from the forehead, for the formation of an artificial nose, they do follow. But in such cases the result is generally in the form of hyperæsthesia: the nose "feels cold"—resulting, probably, from increase I sensibility to the atmosphere; but it resents wounds and other painful injuries as usual; and this often passes away to a considerable degree. In one case, that of a gentleman from America, whom Mr. Gay saw for an incurable ulcer at the base of the great toe, the toe, from about the termination of the metatarsal bone, was devoid of all sensation. The case is worth brief narration. After making every attempt to cure the ulcer, which was about the size of a sixpence and deep, Mr. Gay cut it fairly out with its tissues, and, after liberating the edges by deep lateral incisions, brought the wound together by sutures. It healed, as did the lateral incisions; but it was replaced subsequently, by another ulcer of exactly the same kind and size. The tissues were wholly insensible during the operation. The anæsthesia was occasioned, the patient said, by the removal of the toe nail twelve years before.

In experiments on a sympathetic branch, definite and well known results follow; but the like results do not seem to follow division of its peripheral nerve-filaments, nor do analogous results constantly follow division of the nerve-filaments of the ulnar or pereonal in the event of their division at this part of their respective courses.

Now do not these facts show that, whilst in wounds of nerve-branches of a certain size, and at a certain distance from their peripheral termination, certain functional disorders inevitably follow, the like results do not follow division of their ultimate fibrils? In other words, do they not render it probable that there is, in the periphery of the nervous system, a certain zone in which the insularity in point of conduction, which is so marked in every other part, especially as the nerve-centres, does not exist; and that thus the function of one nerve fibril would instantly be undertaken by another, in case of its being interrupted.

